# Tissue Engineering Challenges for Cultivated Meat to Meet the Real Demand of a Global Market

**DOI:** 10.3390/ijms24076033

**Published:** 2023-03-23

**Authors:** Andressa Cristina Antunes Santos, Denisse Esther Mallaupoma Camarena, Gustavo Roncoli Reigado, Felipe S. Chambergo, Viviane Abreu Nunes, Marco Antonio Trindade, Silvya Stuchi Maria-Engler

**Affiliations:** 1 Department of Clinical and Toxicological Analysis, School of Pharmaceutical Sciences, University of São Paulo, São Paulo 05508-000, Brazil; andressaantunes@usp.br (A.C.A.S.);; 2 Department of Biotechnology, School of Arts, Sciences and Humanities, University of São Paulo, São Paulo 03828-000, Brazil; 3 Faculty of Animal Science and Food Engineering, University of São Paulo, Av. Duque de Caxias Norte, Pirassununga 13635-900, Brazil

**Keywords:** biotechnological, animal-free medium, 3D models, scaffolds, assembly methods

## Abstract

Cultivated meat (CM) technology has the potential to disrupt the food industry—indeed, it is already an inevitable reality. This new technology is an alternative to solve the environmental, health and ethical issues associated with the demand for meat products. The global market longs for biotechnological improvements for the CM production chain. CM, also known as cultured, cell-based, lab-grown, in vitro or clean meat, is obtained through cellular agriculture, which is based on applying tissue engineering principles. In practice, it is first necessary to choose the best cell source and type, and then to furnish the necessary nutrients, growth factors and signalling molecules via cultivation media. This procedure occurs in a controlled environment that provides the surfaces necessary for anchor-dependent cells and offers microcarriers and scaffolds that favour the three-dimensional (3D) organisation of multiple cell types. In this review, we discuss relevant information to CM production, including the cultivation process, cell sources, medium requirements, the main obstacles to CM production (consumer acceptance, scalability, safety and reproducibility), the technological aspects of 3D models (biomaterials, microcarriers and scaffolds) and assembly methods (cell layering, spinning and 3D bioprinting). We also provide an outlook on the global CM market. Our review brings a broad overview of the CM field, providing an update for everyone interested in the topic, which is especially important because CM is a multidisciplinary technology.

## 1. Introduction

Cultivated meat (CM), also known as cultured, cell-based, lab-grown, in vitro or clean meat, has gained prominence in recent years due to increasing societal and industrial interest. CM has arisen as an alternative to help solve environmental, health and ethical issues related to meat product demands [1]. Cellular agriculture is used to produce CM. Its development has been promoted by different concerns: (1) the increase in the global population; (2) the environmental impact of animal agriculture, such as land use, greenhouse gas emissions and impact on biodiversity; (3) animal ethics, including livestock living conditions and slaughter; and (4) the impact of animal agriculture on human health through issues such as animal-borne disease and antibiotic use [2].

Instead of slaughtering animals for food, the meat cultivation process usually starts by obtaining a biopsy and isolating cells. Then, these cells are cultivated to grow and differentiate into skeletal muscle cells. This process can begin with different cell types, but stem cells are an obvious choice due to their ability to proliferate and to differentiate into several lineages [3]. The cells can be obtained from two sources: adult stem cells, which have a limited proliferative capacity, or pluripotent stem cells, which have an indefinite proliferative capacity. The cells usually employed for this application include: (1) muscle satellite cells that are muscle stem cells that can differentiate into myotubes, which is the target cell type; (2) mesenchymal stem/stromal cells (MSCs) that can differentiate into fibroblastic, chondrogenic or adipogenic cell lineages; (3) fibro-adipogenic progenitors (FAPs) that can generate adipocytes and fibroblasts; (4) embryonic stem cells (ESCs); and (5) induced pluripotent stem cells (iPSCs) that can differentiate into any cell type [4].

Two different meat products are expected to be introduced to the market. Unstructured products such as burgers, sausages or nuggets will likely be commercialised first, and then structured products such as a chicken breast or a beefsteak will come later [5]. The manufactured tissue must resemble the in vivo tissue, reproducing morphological and functional characteristics, such as highly aligned muscle fibres and well-distributed fat [6].

**Figure 1:**
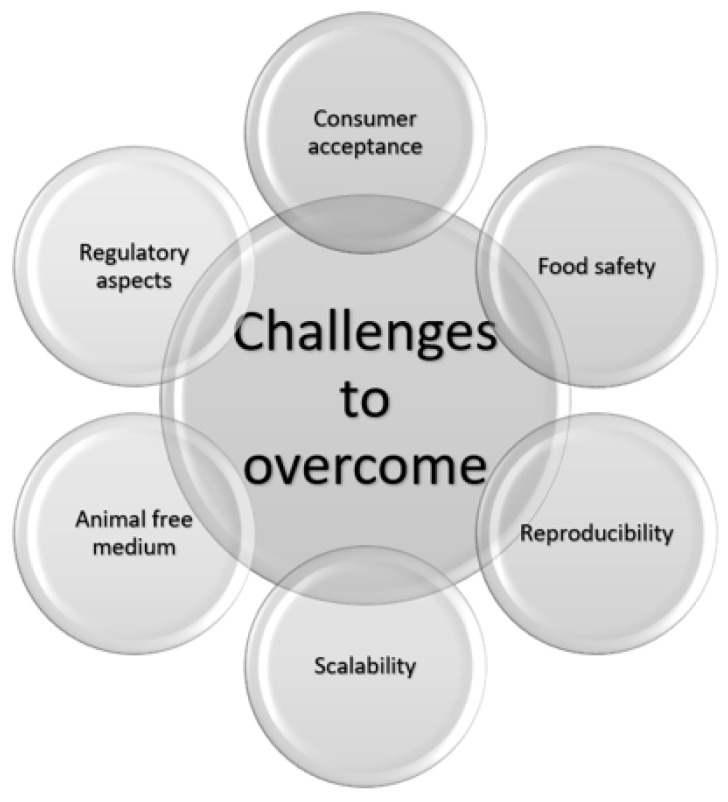
The technological and social challenges that must be overcome before cultivated meat is available on a large scale.

There have been significant advances in the CM field; however, no product is commercially available on a large scale. There are numerous technological and social challenges to this, as shown in Figure 1 [7], that we will address in this review.

## 2. The Cultivated Meat Market

### 2.1. The Global Market

The CM market has grown exponentially in the last few years, producing millions of dollars throughout the world. This growth and the tremendous potential have drawn the interest of several companies [8]. The world’s first cultivated beef burger was revealed in 2013 at a packed press conference in London by Mark Post, who founded the Netherlands-based company Mosa Meat in 2016 [9]. The world’s first CM company was UPSIDE Foods, launched in 2015. Since then, there has been an exponential expansion and dozens of companies in more than 20 countries have been founded. In 2021, at least 21 new companies emerged, which represented significant growth, since until that time there had only been 86 CM companies. Investments have also followed this exponential increase. In 2021, invested capital grew by about 336% from 2020, reaching USD 410 million [10].

Some countries have greater prominence in the global CM market. In April 2022, in the United States, UPSIDE Foods (formerly known as Memphis Meats) raised USD 400 million in Series C funding, a distinguished milestone for the industry [11]. Israel is another important player in this market. In 2021, 36% of CM funding worldwide went to Israeli companies [12]. In 2022, the world’s largest CM consortium raised USD 18 million in government funding for 14 companies and 10 academic laboratories. Important companies such as SuperMeat, Future Meat and Aleph Farms are located in Israel [12,13]. In Europe, the Netherlands, the birthplace of CM, announced EUR 60 million in funding in April 2022 to support the creation of a national cellular agriculture ecosystem as part of the country’s National Growth Fund. Another significant player in this scenario is Spain, which invested EUR 5.2 million in a CM project led by BioTech Foods in 2021 [14].

Over the past two decades, Brazil has been consolidating its position as a major producer of agricultural commodities and related food products as well as a supplier to international markets. It is the fifth-largest country in the world in terms of area and population and the largest in terms of arable land, and it is among the few countries with the potential to increase its agricultural productivity [15]. In the current scenario, the CM field is also extremely attractive and favourable to the emergence of new companies. Brazil is currently the home of a few companies that are involved with CM production [16,17,18,19].

### 2.2. Regulatory Aspects

In contrast to the explosion of the CM market, the regulatory scenario has been developing slowly. Singapore was the pioneer and remains the only regulatory authority in the world to allow the commercialisation of CM [14]. In December 2020, the Singapore Food Agency (SFA) approved the sale of cultivated chicken, in the form of cultivated chicken nuggets (GOOD Meat™), produced by Eat Just. One year later, new products have gained regulatory approval, such as chicken breasts [20].

Progress in the CM industry requires overcoming another big challenge: regulatory approval. It is expected that regulatory approvals will come on a country-by-country basis. In the USA, since 2019 the Food and Drug Administration (FDA) and the United States Department of Agriculture (USDA) have begun to work together to establish a regulatory framework for the industry [21]. Recently, the FDA completed its first pre-market consultation for a human food made from cultured animal cells. While this is not an approval process, it represents a major advance within the global regulatory system [22]. In South Korea, the 2022 National Plan includes official guidance for alternative proteins for the first time. This new guidance could establish the standards for CM [23]. In the Brazilian regulatory environment, GFI-Brazil recently launched a regulatory study that aims to identify possible adjustments in the current regulatory frameworks, supporting this discourse with scientifically based arguments, as well as mobilising the country’s regulatory agents [24].

## 3. Challenges to Overcome

### 3.1. Consumer Acceptance

In general, acceptance of CM is more likely in younger, more educated people who eat meat [25,26,27]. The majority are open to trying CM and purchasing it regularly or even using it to replace conventional meat, but only half are open to paying more for it [25]. Some studies have also shown better acceptance of CM compared with similar food technology innovations, such as genetically modified organisms (GMOs) or insect protein. Nonetheless, to ensure commercial viability, consumer acceptance is essential, and strategies to increase it are necessary. The efforts to achieve this goal focus on lowering costs and reproducing conventional meat characteristics, such as taste, texture and appearance [26].

Consumer reluctance to compromise on the organoleptic properties of conventional meat is the principal industry motivation for providing a product similar in terms of appearance, structure, texture, flavour and nutritional composition. The post-mortem process is the transformation of muscle tissue into meat; this complex process interferes directly with meat properties. Unlike conventional meat, the post-mortem metabolism of CM has not yet been fully described, and basic comparative studies vis-à-vis animal-based meat are needed due to its importance to meat quality.

Conventional meat is commonly processed. The added ingredients aim to improve meat quality in terms of texture and modulating flavour, improving product stability, replacing fat or protein and delivering bioactive compounds. The same principle can be applied to CM during the post-culture period to ensure that CM has comparable properties to animal-based meat products [28].

### 3.2. Food Safety

Among the crucial concerns regarding the willingness to consume CM are flavour, nutrition and safety [29]. Regarding safety, the potential benefits of CM have been highlighted, such as avoiding health issues related to consuming a diet rich in red meat, including zoonotic diseases [30]. The ongoing COVID-19 pandemic caused by severe acute respiratory syndrome coronavirus 2 (SARS-CoV-2) has raised concerns about the use of meat from livestock as well as unconventional animal meat [30,31].

Cultivated cell lines are usually not designated for human consumption. Immortal cell lines could express oncogenes through spontaneous immortalisation or genetic engineering. Thus, it must be confirmed that the food products from these cells do not induce tumourigenicity [29]. Limited evidence suggests that DNA from genetically engineered plant cells can integrate or be transferred into somatic cells or the microflora of the human gastrointestinal tract [32]. Even after confirming the safety of CM, regular monitoring will be needed to avoid contamination and genetic drift. This process can occur over time due to the accumulation of mutations that eventually cause modifications in phenotypes [32].

CM is a disruptive innovation and, as such, is subject to scientific uncertainty. The crucial point lies in finding the optimal timing of market authorisation relative to a better scientific understanding. Although it is worth being cautious to avoid allowing people to consume potentially unsafe products, being overly cautious could impede the benefits of this innovation [30]. The industry’s success is linked to transparency around CM health and safety [26].

### 3.3. Reproducibility

Science is based on existing knowledge and exploring the unknown in pursuit of a significant discovery or paradigm shift. The scientific method consists of precisely controlled and documented experiments using validated reagents. However, the complexity of biology being explored itself, as well as pressures to publish; the focus on novel, positive and impactful results; the use of suboptimal research practices; and the scarcity of research funding results in an undesirable and irreproducible level of scientific data [33]. The reproducibility crisis has been discussed for quite some time. In 2016, a study of 1576 researchers found that 90% agree that there is a crisis related to reproducibility, as more than 70% of them had failed to reproduce other scientists’ experiments. In addition, more than half of these researchers could not repeat their own experiments [34]. Reproducibility is the capacity to replicate an independent finding or the published work’s results using the described data [35]. To improve reproducibility, all required information must be available, and all methods must be reported in detail and without ambiguity [36]. Almost all CM research has been carried out by private companies and their intellectual property is not accessible for refinement and development by the general scientific community [31]. In this way, each company develops their own technology and cultivation process [37]. An alternative to publishing relevant data in this field is funding academic scientific work with a more democratic disclosure of the findings. The lack of publicly available data is the reason why this review contains fewer references than one would typically expect.

### 3.4. Scalability

The ability to scale up CM is crucial for its development. The production process requires an average of 1 × 10^12^–1 × 10^13^ cells to generate ~10–100 kg of CM. Scaling up and scaling out are necessary when a large number of cells is required in biotechnological and bioprocessing industries. Scale-up systems consist of making a component bigger or faster so that it can handle a greater load. Thus, for these systems, the surface area/culture volume is increased progressively as the number of cells is increased. On the other hand, scale-out systems are based on adding more components in parallel to spread out a load. These systems use multiple culture vessels/bioreactors working in parallel.

The cells used for CM production are dependent on anchorage: they must adhere to a surface to remain viable and proliferate. Improving the production process is essential to alleviate the surface-to-volume ratio, to more tightly control critical growth parameters, to optimise dissociation from the growth surface and to ensure an efficient final cell harvest. The strategies to enable a better surface-to-volume balance are: (1) adapt the cells to grow in suspension (anchorage independent) and (2) use suspension culture systems (such as microcarriers), where cells are attached to and proliferate upon carriers that are constantly agitated to remain in suspension [38].

A bioreactor is an essential piece of technology used to scale up the production of cells. This device contains a vessel that supports a biologically active environment, allowing cell growth and development [4]. A bioreactor allocates a significant volume to proliferation, maximises nutrient diffusion and provides mechanical stimulation. The advantages are of great value: it enables large-scale cell culture while also simplifying medium recycling and replacement throughout the proliferation stage. It is possible to control the biological conditions, therefore guaranteeing optimal culture conditions. Oxygen can be introduced by aeration through sparges or upstream to ensure the media is saturated with dissolved oxygen. Sensors monitor the carbon dioxide concentration to maintain the pH at 7.2–7.4. The nutritional support strategy used is usually the fed-batch system, where one or more nutrients can be supplied during the culture period [38].

Some features of a bioreactor need to be accounted for. Mechanical mixing can generate turbulent flow and, consequently, cause cell damage or early apoptosis. The use of primary cells will require a bioreactor with a surface for cells to adhere to or which supports a scaffold. Finally, it is also essential to consider whether a bioreactor could co-culture multiple cell types in the production process. Of course, constant optimisation of bioreactor systems will be necessary for large-scale production to meet industry requirements [4].

### 3.5. Animal-Free Medium

Culture media is crucial for meat cultivation, but it also represents substantial obstacles to CM production. These obstacles mainly consist of cost and ethics, two critical factors for developing and selling a future product. First, the medium represents more than 99% of the expenses in CM production [39]. Currently, the culture media used for CM is the same used to culture cells in the laboratory; it is composed of ingredients of pharmacological grade (high cost). It would be beneficial if the culture medium could be composed of food-grade ingredients (potentially lower cost). Second, cell culture growth is strongly associated with foetal bovine serum (FBS), which is extracted from bovine foetuses. FBS is expensive and, because it is derived from animals, its use is inconsistent with the proposed development of animal-free CM [40]. FBS contains cell attachment factors, micronutrients, trace elements, growth factors and hormones that promote rapid cell growth. A more detailed view of FBS composition is shown in Table 1. Although it originated from an in vivo source, the serum composition can vary dramatically between different batches and also carries the risk of virus or prion contamination for the culture [40,41,42].

Thus, there is an incessant search for the development of a cheap medium, free of animal components and which is capable of sustaining the proliferation and differentiation of bovine satellite cells. Below, we discuss the advantages and limitations of some of these media.

There are some newly developed commercial products that can serve as an FBS substitute. Ultroser G^®^ (Gottingen, Germany) is one of them; although its exact composition is protected by commercial copyright, it contains adhesion and growth factors, vitamins, minerals and hormones [46]. Moreover, the reproducibility of its composition, both quantitatively and qualitatively, guarantees consistent biological activity from batch to batch [47]. Although these FBS substitutes guarantee reproducibility between batches and are easy to use and add to culture media, their use has some disadvantages. They are still relatively expensive, they are not available for sale in several countries and there is no consensus on whether they would allow for culturing any isolated cell type. While [47] reported that Ultroser G^®^ is not a good substitute for FBS for the in vitro culture of bovine embryos, Ref. [48] used it to cultivate bovine satellite cells. However, to date, there does not appear to be a single commercial compound that can replace this serum for all cell types.

Another approach to serum-free media, and the most used and most effective to date, is the development of defined media. Generally, they are designed for specific purposes, to culture a target cell type, and are developed by independent research groups. Here, we highlight four that are relevant for the development of CM.

The first is the Essential 8™ (E8) medium, first described by [49] for the cultivation of human iPSCs cells and, later, commercialised by Thermo Fisher Scientific (Waltham, MA, USA) as a serum-free medium for the cultivation of human stem cells [50]. This medium consists of a DMEM/F12 base in a 1:1 ratio, L-ascorbic acid-2-phosphate (64 mg/L), sodium selenium (14 µg/L), FGF-2 (100 µg/L), insulin (19.4 mg/L), NaHCO_3_ (543 mg/L) and transferrin (10.7 mg/L), TGF-β1 (2 µg/L) or NODAL (100 µg/L). These components will also be found in other serum-free media, making this perhaps a base for the development of other media. The E8 medium has been used to cultivate bovine myoblasts [51]. The authors reported consistently sustained proliferation of myoblasts; however, the number of cells did not reach the level of the control used, DMEM with 20% FBS and 10% horse serum. The authors also studied this medium with the addition of the growth factors EGF and IGF-1, and, unexpectedly, there was no effect on the proliferation, even though this medium contained FGF-2 and insulin, which are known to induce the proliferation of myoblasts [51].

The second medium is an optimisation of the B8 medium [52], which had already been able to reduce the serum requirement in bovine satellite cell cultures by 87.5% [39]. The so-called Beefy-9 or B9 medium seems to perform even better. It consists of a 1:1 DMEM/F12 base with HEPES, insulin (20 µg/mL), L-ascorbic acid-2-phosphate (200 µg/mL), transferrin (20 µg/mL), sodium selenite (20 ng/mL), FGF-2 (40 ng/mL), TGF-β3 (0.1 ng/mL), NRG1 (0.1 ng/mL) and human albumin (800 µg/mL). It could maintain short-term growth comparable to serum-containing medium as well as bovine satellite cell morphology in vitro [39]. The B9 medium has also been evaluated for its ability to maintain the proliferative property of bovine satellite cells. For this, the satellite cells were expanded to confluence in B9 medium and then differentiated in a serum-free differentiation medium, which we discuss later, allowing for visualisation of the formation of multinucleated myotubes [39]. Thus, the B9 medium seems to be promising for culturing bovine satellite cells under serum-free conditions, directly aimed at the development of CM.

The third medium is capable of differentiating bovine satellite cells in a serum-free condition together with the B9 medium. This medium was initially described for the establishment of a system by which mature myotubes could be routinely formed from adult rat satellite cells [53]. It presents as a simple composition of 1:1 Neurobasal/L15 medium with the addition of EGF (0.1 mg/mL) and IGF (0.01 mg/mL). [39] used this to differentiate bovine satellite cells into myotubes in serum-free conditions.

The fourth medium is also aimed at bovine satellite cell differentiation [48]. It is based on the E8 medium, but with the addition of some ligands for receptors identified as upregulated during the initial phase of differentiation of these cells. It has a base of 1:1 DMEM/F12 as well as EGF-1 (10 ng/mL), human albumin (0.5 mg/mL), L-ascorbic acid-2-phosphate (40 mM), sodium selenite (80 nM), NaHCO_3_ (6.5 mM), MEM amino acid solution (0.5%), insulin (1.8 µM), transferrin (135 nM), lysophosphatidic acid (1 µM) and acetylcholine (10 µM). This medium aims to mimic the conditions of serum starvation, a technique used to induce the differentiation of satellite cells, reducing the concentration of serum in the medium to 2%. When compared, both the decrease in serum and the addition of the serum-free medium showed similar results for the gene and protein expression of canonical myogenic markers, indicating that the serum-free differentiation medium induces a myogenic phenotype similar to the technique using serum [48].

The development of defined media for muscle cell proliferation or differentiation has shown promising results in recent years and appears to be the preferred approach. However, there is still another strategy that is promising, less expensive and simpler: the search for natural products of non-animal origin that can replace foetal serum.

Biftek, a Turkish startup, is working on a bacteria-based supplement to replace the use of foetal serum for muscle stem cell growth. Registered under patent US20220098546A1, the substitute is a biological supplement that includes microbiota-derived post-biotics obtained from a fermentation medium of a beneficial microorganism isolated from natural sources. It was able to maintain cell proliferation at rates similar to a conventional medium with FBS [54]. Biftek also highlights that it has achieved a reduction in costs, as its product costs USD 10 per litre to produce [55].

Another research group at Nanyang Technological University in Singapore is working on a substitute for FBS based on okara, a protein- and fibre-rich residue from the production process of certain soy-based products, such as soy milk and tofu [56]. This residue is fermented and a protein hydrolysate is extracted from it, which also contains certain plant growth hormones. This hydrolysate could maintain the proliferation of HEK293 and HepG2 cells and reduced the need for serum in the culture of C2C12 muscle cells. It is currently being tested by startups in Singapore [57,58]. It is also a low-cost product, with a production cost of USD 2 per litre [58].

Benjaminson, Gilchriest and Lorenz [59], in collaboration with the National Aeronautics and Space Administration (NASA), sought substitutes for FBS in the culture medium for the growth of muscle cell explants from goldfish (*Carassius auratus*). They obtained good results with the crude extract from maitake (*Grifola frondosa*), an edible mushroom widely cultivated in Japan, as a source of amino acids, carbohydrates and minerals [60]. They showed a 13.1% increase in the area of fish primary cell explants when using this extract, compared with 13.8% using a standard serum-containing medium [59].

Another product, already used for decades in microbiology for the growth of bacteria, is yeast extract. Ref. [61] investigated it as a serum substitute for bovine skeletal muscle cell culture; it could recover cell growth (metabolism and proliferation) under reduced and serum-free conditions. However, Ref. [62] evaluated this medium in long-term culture and found a significantly lower yield of Vero cells incubated with yeast extract compared with 10% serum.

Based on our current knowledge, we can replace almost all animal-derived substances with recombinant proteins, plant derivatives, fermentation derivatives or synthetic compounds [63]. The culture media that use these techniques are presented in Table 2. Even at this stage of our knowledge, there is still no single strategy for formulating a serum-free medium that works efficiently for every cell type in every situation [5]. Therefore, the development of an animal-free medium is still a challenge.

## 4. Three-Dimensional Models for Cultivated Meat Production: Technological Aspects

**Figure 2:**
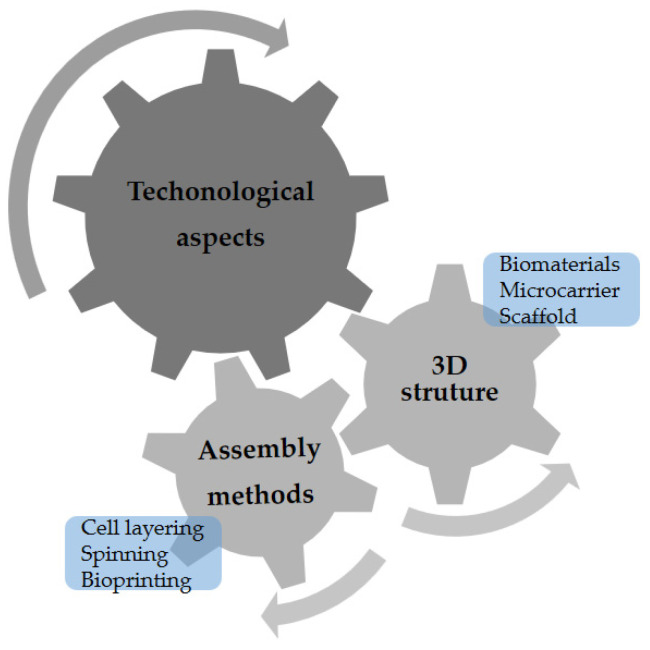
The technological aspects that must be considered for cultivated meat production.

Initially, in vitro studies maintained the cells in a two-dimensional (2D) culture, which allowed for an understanding of the biological mechanisms underlying cell functions, such as migration and differentiation. Over time, interdisciplinary improvements were made, and the application of biomaterials in culture was essential for the creation of a three-dimensional (3D) environment. It is possible to simulate variable and complex topographies in which cells can more closely mimic the behaviors of their in vivo environments [64,65]. Figure 2 shows some technological aspects that must be considered for CM.

Tissue construction aiming at successful recreation must mimic an extracellular matrix (ECM), including the composition, the physical properties and the technique for building a structure, all of which will affect the mechanical characteristics of the generated tissue [66]. It is also important to bear in mind that each tissue has a characteristic ECM, and different types of scaffolds can provide different cell targeting and differentiation outcomes [67]. Different scaffold architectures can be achieved with existing techniques like porogen leaching, gas foaming, freeze-drying, electrospinning, 3D printing [68], sol-gel transition of gelatine [69] and 3D bioprinting (3DP) [70], among others.

**Figure 3:**
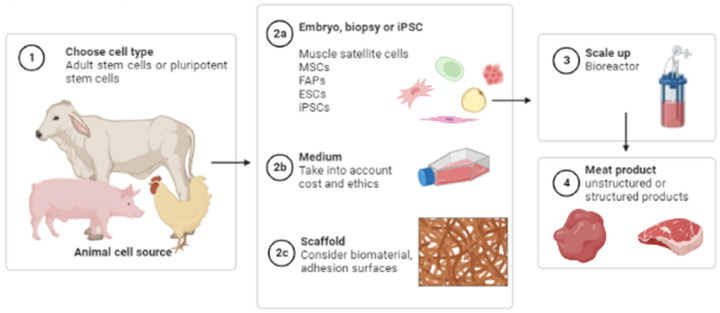
A general workflow for cultivated meat production.

CM is obtained through a process called cellular agriculture, which is based on tissue engineering principles [31]. Tissue engineering requires three technical components: cells, signals and scaffolds. In practice, it is first necessary to choose the best cell source and type. Second, a biocompatible tissue scaffold should be selected to provide structural support to those cells so they can proliferate and differentiate. Finally, but equally important, the necessary nutrients and small molecules must be provided; they will serve as external signals necessary for cell development (Figure 3).

### 4.1. Biomaterials

The biomaterials used in tissue engineering are commonly classified according to their origin as natural or synthetic. The natural biomaterials include chitosan, hyaluronic acid, fibrin, alginate, elastin, keratin, poly(hydroxybutyrate) (PHB) and decellularised extracellular matrix (dECM), among others. Some synthetic materials include polyglycolic acid (PGA), polylactic acid (PLA), poly DL-lactic co-glycolic acid (PLGA), polycaprolactone (PCL) and polyethylene glycol (PEG) [64,71]. Table 3 presents some examples of scaffolds with different compositions generated via different techniques.

The ideal material should be unlimited, biocompatible—to which cells can bind and interact with the extracellular proteins necessary to form the tissue—non-toxic and edible, which is one of the biggest challenges. Collagen is an animal-derived material used in a mixture with Matrigel and can be considered as a matrix or support. It enables differentiating myoblasts to align, compact and form a muscle fibre [83]. Plant-derived or synthetic polymers are an alternative to avoid using proteins from animal sources. The main difference between decellularised plant material and material of animal origin is the presence of ECM proteins. These proteins represent a mix of functional molecules such as collagen, fibronectin, glycosaminoglycans and a variety of growth factors that can influence cell development [84,85]. The absence of these molecules influences cell fixation and proliferation. Due to its biocompatibility, cellulose has arisen as a promising candidate for cell adhesion improvement [84]. However, other options are also viable: [86] showed successful myoblast cultivation in agarose, gellan and a xanthan–locust bean gum blend (XLB) as support materials with pea and soy protein additives.

As CM is designed for human consumption, compounds accepted by the FDA are favoured. The supply of these natural sources can guarantee availability for large-scale production. Among those already known are alginate, chitins and cellulose, which are widely used in food applications. Table 4 shows possible alternative substances to be used in the construction of potential scaffolds, using some FDA-accepted and listed substances that are generally already used in the food industry as additives [87,88,89]. Note that agarose, gellan, xanthan gum, locust bean gum and pea and soy protein have already been used to construct scaffolds. Hence, they could be useful for CM production.

### 4.2. Microcarriers

Microcarriers have been used for quite some time in animal cell culture and they should be able to adapt to CM without major obstacles [108]. Although cell attachment is often a complex and empirical function, the interdependent factors are hydrophilicity, surface topography, net surface charge, charged group density, curvature and shear rates [28].

Cells attach and grow by apposition in microcarriers, which are beads ordinarily having a diameter of 100–200 μm [109]. Microcarriers differ in their physical properties such as size, porosity, rigidity, density and surface chemistry [110]. They can be made of synthetic or natural polymers. As mentioned, these microcarriers are applied to large-scale bioprocesses allowing for efficient proliferation of anchor-dependent cells. The advantages of this method are: easy production of large quantities of material and compatibility with various bioreactors and efficient proliferation of adherent cells. The disadvantages are the costs and potential inedibility [31]. For those that cannot become an integrated part of final product, the cells can be harvested from microcarriers by changing temperature, or through electronically induced shape change [109]. There is usually a significant cell/tissue yield lost independent of the dissociation process because the cell detachment is incomplete; this loss directly impacts the production efficiency and costs [110]. On a smaller scale, other alternatives to microcarrier cultures are being considered [111], such as spheroids [112,113], organoids [114] or single-cell suspension cultures [115].

### 4.3. Scaffolds

Scaffolds are 3D structures made to resemble the in vivo environment. These porous materials provide mechanical support and allow for an integrated network. Scaffolds are usually used to aid the differentiation step because they enable cells to adhere and mature into an edible meat product. Depending on the model type, scaffolds can grant potential vascularisation and spatial heterogeneity, essential features that the improve texture and structure of the final product, making it more like conventional meat [31].

Because CM is an edible product, the tissue scaffolds should be biodegradable and non-toxic. However, in some cases, they may be designed to be degraded or removed before consumption [4]. The scaffolds may also have appropriate mechanical properties, including strength, thickness, stiffness, pore size, texture and architecture [31]. For example, porosity directly influences media perfusion, and tissue maturation for a similarity with conventional meat scaffolds also need to support tissue maturation beyond a thickness of 1 cm [116]. Other properties such as nutritional value, thermal stability, non-allergenicity, non-toxicity and the ability to improve organoleptic properties are important items to consider when choosing the best scaffold depending on the final desired product [31].

Currently, there is no commercially available scaffold for CM that is free of animal biomaterials, although a scaffold could be derived from natural, synthetic or composite biomaterials. Natural materials can be derived from animal sources, such as collagen, fibrin and hyaluronic acid, or from plant origin, like alginate, decellularised plant materials and cellulose. Other sources of scaffold materials include chitosan from crustaceans, yeast or fungi and fungal mycelium. Synthetic polymers include a range of polyesters (polyamide and polyethylene). Scaffolds made of these materials can be food safe. Synthetic polymers such as PLA and PLGA are degraded by chemical hydrolysis to generate products like lactic acid and glycolic acid, which are considered safe in food [116].

Textured soy protein (TSP) is an important potential scaffold to consider. It is a porous, food-grade, inexpensive by-product of soybean oil processing that has great nutritional value and high protein content in addition to improving the final texture of the product. Due to its characteristics, it is commonly used in plant-based meat substitutes and does not require major modifications; hence, it is highly applicable for mass production. TSP can be adapted to various sizes and shapes, thus facilitating implementation in cultivation processes—for example, in bioreactors [117].

Other materials have characteristics that have attracted attention for scaffold production. A group in Korea fabricated microspheres of gelatine (GMS) to use as a scaffold with a high surface-area-to-volume ratio for CM. Gelatine is an edible material capable of promoting cell adhesion through its tripeptide Arg-Gly-Asp (RGD) sequence. In addition to being a collagen-derived natural polymer, its other advantages include high biocompatibility, biodegradability and processability [118]. Meat analogues with a suitable scaffold made of gelatine fibres can be produced through dry-jet wet-spinning, producing 3D aligned tissues. Aortic smooth muscle cells and skeletal muscle myoblasts have been cultivated in gelatine fibre scaffolds, which are safe and edible materials [119].

## 5. Assembly Methods

Functional tissues require characteristics such as mechanical stiffness and chemical and surface properties for desirable cellular interactions to trigger cell responses. Because scaffolds are important for mimicking the complex spatiotemporal distribution of in vivo tissue, some assembly methods used in tissue engineering to manufacture such structures have been developed [6].

### 5.1. Cell Layering or Self-Assembly

Layer-by-layer (LbL) assembly is a highly adjustable and simple multilayer self-assembly technique. It is possible to produce multilayer coatings with a specific architecture and composition from an extensive catalogue of available materials for several biomedical applications [120]. This production process is fast and scalable, and it can manufacture highly dense, multicellular and textured tissues in normal culture plates without a bioreactor. A bioreactor is necessary if the final product needs to be thicker [119].

There are three basic methods for cell layering: stacking cell sheets, rolling a cohesive tissue sheet and in situ deposition of cell-laden biomaterials. The first one uses a temperature-responsive polymer-coated culture dish to form a multi-layered tissue. The second method consists of wrapping a whole piece of a thin tissue sheet around a tubular support and culturing until tissue fusion. For the third approach, a handheld apparatus is used to deposit cell-laden biomaterials [6].

Biomaterials are used to promote or prevent cell adhesion, to maintain or direct cellular phenotypes, and to provide 3D structures for cell culture or co-culture. The range of biomaterials applied for LbL assembly include biomolecules, polyelectrolytes, particles and colloids, among others [120]. The successful co-culture of myoblasts and preadipocytes has already demonstrated the feasibility of this approach for building meat-like tissues of any size and thickness. Scaffolds do not need to be used because the cells produce their own ECM that is preserved and makes robust sheets [119].

### 5.2. Spinning

Spinning refers to a manufacturing process for creating fibre-shaped materials and can be applied in various manufacturing fields, including tissue engineering [121]. This material is a potential choice for in vitro tissue production because it produces highly aligned structures with long lengths and flexibility, features that produce functional and morphological characteristics. Among the spinning methods, wet spinning and electrospinning are suitable for this [6]. Wet spinning is commonly used to produce fibres with micron diameters by using polymers dissolved in non-volatile or heat-unstable solvents. Electrospinning enables the production of nanofibers that can meet the functional requirements of tolerating high temperatures and demonstrating strong absorption for filtration [122].

Wet-spun fibres allow for cell adhesion and proliferation into highly oriented porous structures. For this approach, diverse biomaterials can be used as polymers, such as PLGA, chitosan and alginate [121]. Cells can be mixed with biopolymers and laden or encapsulated within the polymer through microfluidics devices, forming cell-laden fibres. It is possible to assemble larger scaffolds or tissues with this methodology. The main advantage of this method for CM production is the ability to modulate the thickness, shape and mechanical rigidity of the fibres through microfluidic channels [6].

Electrospinning is based on the use of electrical forces to produce fibres, which provide a large surface-area-to-volume ratio, thus improving cellular development. Biopolymers for cell electrospinning can also be natural or synthetic polymers [123]. Electrospun scaffolds are also very interesting for CM production because this technique generates alignment cues that guide the uniaxial alignment of seeded cells. Ref. [124] demonstrated that even after electrospinning, myoblasts on fibrin scaffolds exhibited a uniform distribution, and they continued to proliferate and differentiate.

### 5.3. Bioprinting

3DP is a promising tissue engineering technique for simulating the structural characteristics of meat. The advantages of 3D bioprinting are the ability to control the structure and composition of a product in addition to its potential scalability (Kang et al. 2021). 3DP is an advanced additive manufacturing platform that allows for the pre-defined deposition of cells, biomaterials and growth factors. It is based on computer-aided design and manufacturing (CAD/CAM), which customises the layer-by-layer printing process with a high level of flexibility and reproducibility. 3DP is an emerging technology that has attracted increased attention in the last few years in the food field due to its applicability for sustainably manufacturing customised products with intricate shapes and textures [125]. It also allows for improving the nutritional profile and sensorial values of the product [126].

The 3DP production process allows for the deposition of materials or inks in a layer-by-layer fashion to generate complex 3D structures that resemble laboratory cultured cells [126]. The most-used 3DP methods are extrusion, inkjet printing, binder jetting and bioprinting. For meat product fabrication, the extrusion method is commonly used to print the 3D structures [125].

Bio-inks consist of cells, biomaterials and other molecules such as growth factors. The medium for the cell suspension contains polymer crosslinkers, such as CaCl_2_, thrombin, salt (NaCl), gelatine and fibrinogen. Biomaterials, such as melt-cure polymers, hydrogels or dECM, are utilised as scaffolds in bio-inks to provide an appropriate microenvironment for cell adhesion, migration and differentiation [1,6,127]. Therefore, 3DP provides the possibility for reshaping the structure of conventional meat: the structure can be designed in such a way that raw materials can be thoroughly mixed and organised. With this process, it is possible to fabricate flexible artificial vessels and to control the graininess and toughness of the final product, to ensure that it is similar to conventional meat [108].

Printed constructs enable nutrient diffusion and enhance porosity. Researchers have described successful printing of engineered tissues, including skeletal muscle [128,129,130]. Obviously, 3D-printed CM has benefited from advances made in the tissue engineering field. Although it does not need a complex vascular system like in natural tissue, more research is required to improve printable, non-animal materials and potentially edible scaffold compositions.

Ianovici et al. (2022) [1] tested two 3D-printed scaffolding compositions, not derived from animal biomaterials and enriched with plant proteins, for bovine satellite cell cultivation. They evaluated mixtures of pea protein isolate (PPI) and soy protein isolate (SPI) with RGD-modified alginate suitable for flexible 3DP and cell cultivation. They observed bovine satellite cell attachment, spreading, maturation and differentiation. They applied extrusion using an edible, removable agar support bath. PPI-enriched bio-inks allowed for cellular bioprinting. 

Kang et al. (2021) [131] produced the first whole-cut CM-like tissue. It was composed of three types of primary bovine cells (satellite cells, adipose-derived stem cells and endothelial cells). The authors used tendon-gel-integrated bioprinting (TIP) to fabricate cell fibres. They then modelled the subsequent cell differentiation into the structure of real meat. When assembled, it mimicked the histological structures of a real steak. Despite its good appearance, the meat-like tissue was very small and not edible, indicating that more research is needed for improvement. Although 3DP is likely to achieve a final product with a thickness close to that of real meat, it might be less amenable to the scaling required to achieve CM production.

## 6. Conclusions

Meat is exsanguinated and aged musculoskeletal tissue—comprising skeletal muscle, connective tissues, bone, blood vessels and nerves—that is derived from biochemical reactions triggered by lack of oxygen following the slaughter of the animal [28,132]. CM is an emergent and disruptive technology in cellular agriculture that aims to reproduce, ultimately, all of the organoleptic properties of meat. Currently, most CM is muscle protein made solely from muscle fibres [133]. An imitation of the complexity of conventional meat needs to have a 3D structure and multiple cell types, but the technology for this assembly process has not yet been developed (Table 5). The expectation is that, initially, unstructured products (burgers, sausages and nuggets) will be produced and marketed.

The future of CM is uncertain, but it already has the potential to become a significant part of the meat industry in the coming years. Many see it as a sustainable alternative to conventionally produced meat. Since 1931, when Winston Churchill made the remarkable prediction that it would be possible to grow chicken breasts and wings without the ‘absurdity of growing a whole chicken’ [135], tissue engineering has increased tremendously and in the future will play more and more of a role in the food industry. However, there are still technical and economic challenges to overcome, such as scalability, cost and regulatory approval, before it can be produced at scale and sold at prices competitive with conventional meat. Additionally, there may be cultural hurdles to overcome before CM is widely accepted by consumers. Overall, the future of CM is likely to be shaped by a combination of scientific and technological progress, changing consumer preferences and regulatory developments. The industry is expected to grow in the coming years: investments and partnerships from major food companies and startups should shorten the wait time for CM to hit market shelves.

## Figures and Tables

**Table 1:** Constituents of foetal bovine serum.

Component Category	Components	Quantity (When Specified)
Proteins	Albumin Fibronectin Globulins Protease inhibitors Transferrin	40–80 g/L overall
Hormones	Insulin Glucagon Corticosteroids Vasopressin Thyroid hormones Parathyroid hormone Growth hormone Pituitary glandotropic factors Prostaglandin	------
Growth factors	EGF PDGF IGF-1 and 2 FGF IL-1 IL-6 TGF-β1 NGF	1–100 μg/L overall
Lipids	CholesterolLinoleic acid Phospholipids	2–10 g/L overall
Carbohydrates	Fructose Galactose Glucose Glycolytic metabolites Mannose Ribose	1–2 g/L overall
Vitamins	Vitamins A, C and E, and the B group	10 μg/L to 10 mg/L overall
Minerals	Ca, Cl, Cr, Cu, F, Fe, I, K, Mn, Mo, Na, Ni, Se, Sn and Zn	0.79 μg/L to 3.57 g/L overall

Adapted from [5,43,44,45].

**Table 2:** Serum-free media: composition and uses.

Medium	Composition	Use	Reference
E8	DMEM/F12 in a 1:1 ratio, L-ascorbic acid-2-magnesium phosphate (64 mg/L) with sodium selenium (14 µg/L), FGF-2 (100 µg/L), insulin (19.4 mg/L), NaHCO_3_ (543 mg/L) and transferrin (10.7 mg/L), TGFβ1 (2 µg/L) or NODAL (100 µg/L)	Growth of primary bovine myoblasts	Kolkmann et al. (2020) [51]
Beefy-9	DMEM/F12 in a 1:1 ratio with HEPES, insulin (20 µg/mL), L-ascorbic acid-2-phosphate (200 µg/mL), transferrin (20 µg/mL), sodium selenite (20 ng/mL), FGF-2 (40 ng/mL), TGF-β3 (0.1 ng/mL), NRG1 (0.1 ng/mL) and human albumin (800 µg/mL)	Bovine satellite cell growth	Stout et al. (2022) [39]
McAleer differentiation medium	Neurobasal/L15 1:1 with addition of EGF (0.1 µg/mL) and IGF (0.01 µg/mL)	Differentiation of bovine satellite cells	McAleer et al. (2015) [53]; Stout et al. (2022) [39]
Messmer differentiation medium	DMEM/F12 1:1 with EGF-1 (10 ng/mL), human albumin (0.5 mg/mL), L-ascorbic acid-2-phosphate (40 µM), sodium selenite (80 nM), NaHCO_3_ (6.5 mM), MEM amino acids (0.5%), insulin (1.8 µM), transferrin (135 nM), lysophosphatidic acid (1 µM) and acetylcholine (10 µM)	Differentiation of bovine satellite cells	Messmer et al. (2022) [48]
Benjaminson Maitake medium	MEM-Hanks’ with maitake extract in a ratio of 1:9	Growth of fish muscle cell explants	Benjaminson, Gilchriest and Lorenz (2002) [59]
Andreassen yeast medium	DMEM with 10 mg/mL of yeast extract	Maintenance of primary skeletal bovine cells	Andreassen et al. (2020) [61]

Note: We could not describe media that are under patent or that are embargoed.

**Table 3:** Chemical compositions and techniques used to construct scaffolds.

Composition	Technique/ Feature	Application	Reference
Poly(3-hydroxybutyrate), poly(3-hydroxybutyrate-co-3-hydroxyvalerate)	Electrospinning	Bone scaffolds	Sombatmankhong et al. (2007) [72]
Chitosan/hydroxypropylated oxide/ethylene glycol functionalised nanohydroxyapatite	Nanocomposite	Bone tissue	Depan et al. (2011) [73]
Fibrin	Microthread extrusion	Skeletal muscle regeneration	Page et al. (2011) [74]
Cellulose/fibronectin	Spin coating	Skeletal muscle myogenesis	Dugan et al. (2013) [75]
Hydroxyapatite/polyethylene glycol maleate citrate/polyethylene glycol diacrylate	Hydrogel	Orthopaedics	Gyawali et al. (2013) [69]
Hyaluronic acid/chitosan/plasmid-DNA nanoparticles	Nanoparticle incorporation	Cartilage tissue	Lu et al. (2013) [76]
Alginate	Oligopeptide modification for lyophilised hydrogel generation	Skeletal muscle injures	Wang et al. (2014) [77]
Polylactic acid/collagen	Electrospinning	Tendon reconstruction	Sensini et al. (2018) [78]
Polyethylene glycol macromere/laminin	Hydrogel	Neural stem cell culture systems	Barros et al. (2019) [79]
Polyethylene glycol	Hydrogel	Bone marrow	Wei et al. (2020) [80]
Skeletal muscle–derived decellularised extracellular matrix (dECM)/IGF-1	dECM hydrogel and poly L- lactic acid	Skeletal muscle regeneration	Lee et al. (2020) [45]
Polyurethane/gelatine	Electrospinning	Skin regeneration and repair	Sheikholeslam et al. (2020) [81]
Poly L-lactic acid/gelatine	Electrospinning	Schwann cells	Niu, Stadler and Fu (2021) [82]

**Table 4:** Examples of substances recognised by the Food and Drug Administration (FDA) as safe for food industry use that have been used to construct scaffolds.

Biomaterial	Food Industry Use	Tissue Engineering			
		Technique	Cell Culture	Application	Reference
Chitosan	A1077 ^4^	Freeze- drying	Fibroblasts (NIH3T3)	Potential for tissue regeneration	Nwe, Furuike and Tamura (2009) [90]
Beta-glucan soluble fibre ^1^	Component of cell wall material in barley and oats ^5^	Electrospinning	L6 myoblasts (NCCS, Pune)	Potential skin scaffold material	Basha, Sampath Kumar and Doble (2017) [91]
Starches	EM, St, Tck ^6^	Suspension, free- drying	Cancer cell line (HepG2)	Matrix for culturing living cells	Prasopdee et al. (2021) [92]
Psyllium husk ^1^	St, FS, 0.5% by weight in frozen desserts ^7^	Freeze- drying	L929 fibroblast	Potential macroporous scaffold in TE	Poddar et al. (2019) [93]
Powdered cellulose ^1^	Aa, Ba, EM, Ga, H, St, Tck ^6^	Decellularisation	NIH-3T3 stably expressing GFP-actin	Potential macroporous and fibrous scaffold in TE	Bar-Shai et al. (2021) [94]
Guar gum ^1^	EM, St, Tck ^6^	Hydrogels/freeze-drying	Human keratinocytes (HaCaT)	Scaffolds desirable for soft TE	Indurkar et al. (2020) [95]
Pectin ^1^	EM, Gg, Ga, St ^6^	Hydrogel/crosslinking/electrospinning	Human bone marrow–derived MSCs	Potential scaffold for vascular TE	Li et al. (2019) [96]
Locust bean gum ^1^	EM, St, Tc ^6^	Cryogels/freeze- drying	NIH-3T3 cells	Macroporous scaffold for cartilage and other soft tissue	Bektas et al. (2021) [97]
Hydroxypropyl methyl cellulose ^1^	Ba, EM, Ga, St, Tc ^6^	Crosslinking/freeze-drying	Human Saos-2 osteoblast-like cells	Potential scaffold for bone graft for alveolar bone regeneration	Feroz and Dias (2021) [98]
Arabinoxylan ^2^	Binder, Gg, Txz, St, Tck, EM ^8^	Freeze- drying	MC3T3-E1 cell lines	Regenerate fractured bone	Khan et al. (2021) [99]
Alginate ^2^	EM, FAg, FE, FAd, PAd, St, Tck, Sag, Txz ^3^	Hydrogel/crosslinking	C2C12 murine myoblasts	Potential to regenerate skeletal muscle	Aparicio-Collado et al. (2022) [100]
Acacia (gum arabic) ^2^	Ba, carrier, EM, Ga, St, Tck ^6^	Crosslinking/gel	MSCs from human placenta and IVD	Potential candidate in applications in TE	Rekulapally et al. (2021) [101]
Agarose	In agar (Ba, carrier, EM, Ga, Gg, H, St, Tck) ^6^	Hydrogels	Murine myoblast C2C12 cell line	Polysaccharide–protein scaffolds as potential candidates for cultured meat	Wollschlaeger et al. (2022) [86]
75Gellan	EM, FoAg, St, Tck ^6^				
Xanthan gum	EM, FoAg, ST, Tck ^6^				
Locust bean gum					
Pea protein					
Soy Protein					
Polyvinyl alcohol	Ga, Tc ^6^	Freeze- drying	Not used	Potential to be applied in the field of TE that demands high strength	Sun et al. (2022) [102]

Aa—anticaking agent; Ba—bulking agent; ECM—extracellular matrix; EM—emulsifier; FAd—formulation aid; FAg—firming agent; FE—flavour enhancer; FoAg—foaming agent; FS—substances permitted as optional ingredients in a standardised food; Ga—glazing agent; Gg—gelling agent; H—humectant; IVD—intervertebral disc; MSC—mesenchymal stem cells; PAd—processing aid; Sag—surface-active agent; St—stabiliser; Tck—thickener; TE—tissue engineering; Txz—texturiser. ^1^ The FDA has identified the following isolates or synthetic non-digestible carbohydrates as meeting the definition of dietary fibre [103]. ^2^ The FDA intends to propose that the following non-digestible carbohydrates be added to the definition of dietary fibre [103]. ^3^ [87]. ^4^ A1077 fungal chitosan from *Aspergillus niger* is a processing aid for a number of purposes including as a fining and clarifying agent in the manufacture of wine, beer, cider, spirits and food-grade ethanol [104]. ^5^ [88]; FAO (2022). ^6^ [105]. ^7^ [106]. ^8^ Corn bran arabinoxylan (BFG) is proposed for use as a formulation aid at a maximum use level of 3% and a good source of fibre at a maximum use level of 3.8 g/serving in a variety of food categories. [107].

**Table 5:** The outlook of cultivated meat (CM) production in recent publications.

Title	Conclusions	Authors
Cell sources for cultivated meat: applications and considerations throughout the production workflow	The development of highly proliferative, multipotent livestock cell sources is a crucial technical challenge in the effort to scale up CM production for commercial sale. Further advancements to develop immortalised off-the-shelf cell lines will be needed to reach the necessary scale and cost for commercial production and sale of CM products.	Reiss et al. (2021) [4]
Bioengineering outlook on cultivated meat production	On a global scale, the CM industry is still at the proof-of-concept stage. The CM industry will need to overcome its cost of production, primarily associated with metabolic inefficiency, shear-induced cell damage and low growth rates. It is also necessary to improve the proliferative capacity of the cells and to create immortalised cell lines of different livestock species.	Pajčin et al. (2022) [31]
Considerations for the development of cost-effective cell culture media for cultivated meat production	Clearly, the design of culture media to achieve scalable, low-cost and high-quality CM products remains a complex challenge. Continuing research should be focused on developing an understanding of how the molecular mechanisms controlling muscle cell growth and differentiation can be simulated via more affordable and ethical means.	O’Neill et al. (2021) [5]
Scaffolding biomaterials for 3D cultivated meat: prospects and challenges	Although the development of appropriate scaffolds for CM is challenging, it is also tractable and provides novel opportunities to customise meat properties. Future research will provide scaffolds capable of supporting the growth of high-quality meat while minimising production costs.	Bomkamp et al. (2022) [111]
Scaffolds for the manufacture of cultured meat	So far, the scaffolds used in CM research are predominantly collagen and gelatine, which are derived from animals. While many materials and processing techniques have great potential, the major challenge faced by this field is the development of a vascularised, perfusable scaffold that can be employed to provide some form of structure to CM such that meat products can mimic steak and strips.	Seah et al. (2021) [3]
Integrating biomaterials and food biopolymers for cultured meat production	CM faces significant impediments to market feasibility. This is due to fundamental knowledge gaps in producing realistic meat tissues via conventional tissue engineering approaches, as well as translational challenges in scaling up these approaches in an efficient, sustainable and high-volume manner.	Ng and Kurisawa (2021) [28]
Consumer acceptance of cultured meat: an updated review (2018–2020)	Consumers are particularly open to the concept of CM; they mostly identified animal- and environment-related benefits as drivers of this. In the long-term, objections based on neophobia and norm violation will decrease, and widespread acceptance will depend in large part on the price and flavour of CM.	Bryant and Barnett (2020) [26]
Brazilian consumers’ attitudes towards so-called “cell-based meat”	Although they would not be willing to pay more for CM than for conventional meat, younger respondents have the highest willingness to consume it. The answers of 4471 respondents revealed that 46.6% of them thought CM was promising and acceptable and more than 66% were willing to try it.	Chriki et al. (2021) [134]

## Data Availability

The research data described in the manuscript is available.
